# Intracranial and Spinal Tuberculosis: A Rare Entity

**DOI:** 10.7759/cureus.20787

**Published:** 2021-12-28

**Authors:** Sara Rehman, Anis U Rehman, Muhammed A Naveed, Aamer Iftikhar

**Affiliations:** 1 Radiology, Shaukat Khanum Memorial Cancer Hospital and Research Centre, Lahore, PAK

**Keywords:** pott's disease, tuberculoma, spine, cns, tuberculosis

## Abstract

CNS tuberculosis has a broad spectrum of disease patterns and a high risk of complications and mortality. We present a case of a 36-year-old man who was diagnosed with neurotuberculosis with intracranial and spinal tuberculomas, meningitis, and spondylodiscitis. The patient was a known case of sarcoidosis and was being managed on corticosteroids. His presenting complaints were headache, low-grade fever, severe backache, lower limb weakness, and one episode of altered sensorium. The initial diagnosis was based on imaging findings, which were confirmed with positive cerebrospinal fluid (CSF) culture for Mycobacterium tuberculosis. Imaging and clinicopathological correlation enables early diagnosis and treatment and prevents permanent neurological sequelae.

## Introduction

CNS involvement by tuberculosis (TB) is considered the most devastating form of extra-pulmonary involvement of disease, comprising 10% of all TB cases [1]. It carries a risk of permanent neurological sequelae and mortality. CNS TB usually results from hematogenous spread, while direct spread from intra- or extra-cranial focus is rare. Neurotuberculosis can present with meningeal form, which includes leptomeningitis and pachymeningitis, while the parenchymal forms include tuberculoma, tubercular cerebritis and abscess, tubercular rhombencephalitis, and tubercular encephalopathy. Other forms include osseous and non-osseous spinal/spinal cord TB, subdural/epidural abscess, and calvarial TB involving the CNS with direct or indirect pathways [2]. Imaging plays a pivotal role in the accurate and timely diagnosis of different forms of disease and thereby reducing morbidity and mortality.

## Case presentation

A 36-year-old man was diagnosed with sarcoidosis when he presented five years ago with complaints of cough, exertional dyspnea, weight loss, and mediastinal lymphadenopathy on thoracic CT scan. EBUS (endobronchial ultrasound) guided fine needle aspiration was performed from the right lower paratracheal lymph node, which showed non-caseating granulomatous inflammation. The serum calcium level was 10.2 mg/dL. Prednisolone 5 mg was initially started with dose escalation to 30 mg daily. The patient was on regular imaging and biochemical follow-up after which the steroids were gradually tapered down over a period of six months and then eventually stopped. Three months later, he relapsed with a dry cough, fever, and weight loss. His chest radiograph showed perihilar airspace disease. He was diagnosed as a relapse of sarcoidosis with stage 2 disease and was restarted on prednisolone 20 mg twice a day with subsequent improvement in pulmonary symptoms. Subsequently, he presented to the emergency department with complaints of headache, low-grade fever, severe backache, and lower limb weakness. He had one episode of altered sensorium and confusion. Serum calcium was 8.56 mg/dL and serum creatinine was 1.13 mg/dL.

**Figure 1:**
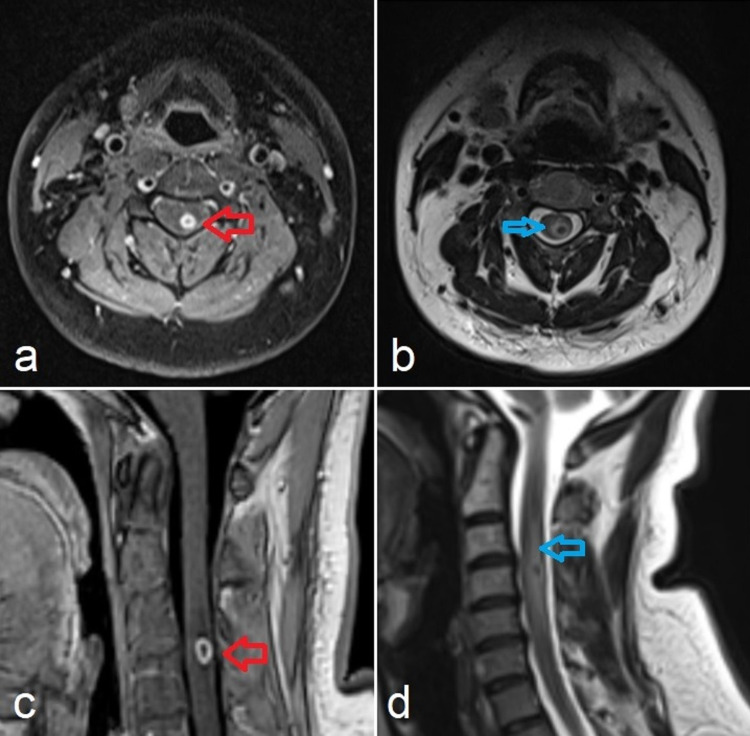
MRI images of the cervical spine, (a) axial and (c) sagittal T1WI post-contrast sequences, shows a ring-enhancing lesion (red arrows) in spinal cord opposite C4 vertebral body with surrounding peripheral edema, blue arrows in T2WI axial (b) and sagittal (d). MRI, magnetic resonance imaging; T1WI, T1-weighted image; T2WI, T2-weighted image

Magnetic resonance imaging (MRI) of the spine with contrast was performed that showed an 8-mm rim-enhancing intramedullary lesion in the left half of cervical spinal cord opposite C4 vertebra with surrounding edema (Figure 1).

**Figure 2:**
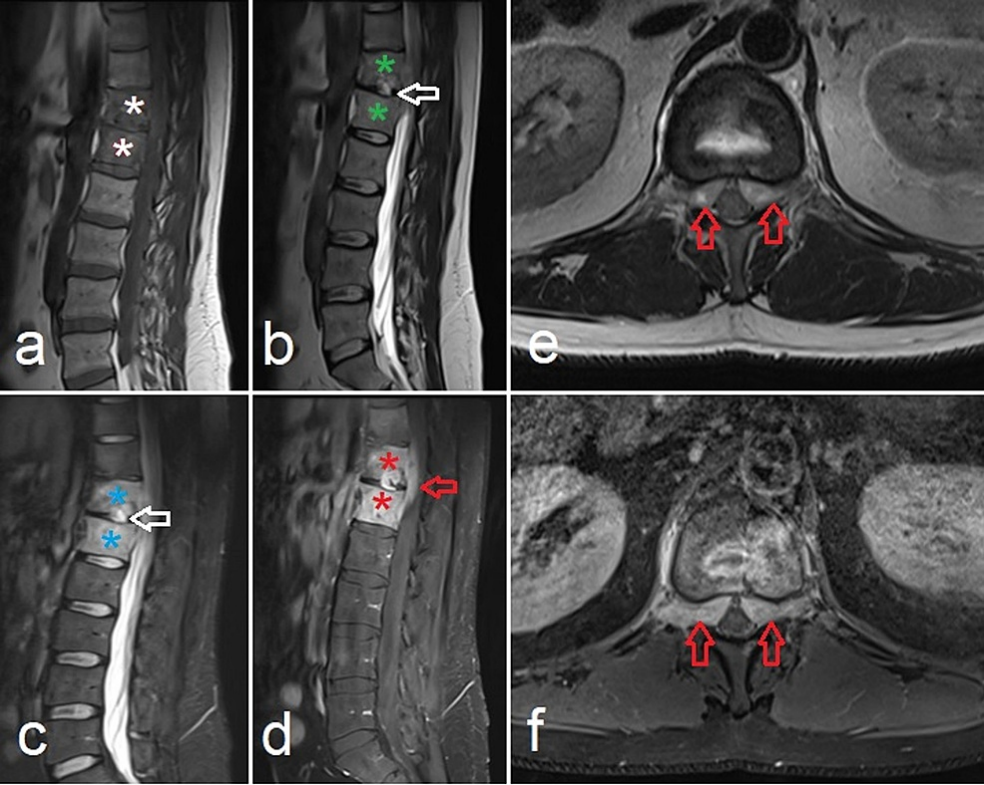
MRI of the lumbar spine shows T1WI hypointense (white asterisks in a), T2WI intermediate (green asterisks in b), STIR hyperintense (blue asterisks in c) signals involving T12-L1 vertebral bodies. Hyperintense signal is also seen in the intervertebral disc protruding into the inferior endplate of the T12 vertebral body, suggesting discitis (white arrow in b and c). T1WI post-contrast sagittal (d) and axial (f) sections show heterogeneous post-contrast enhancement of T12 and L1 vertebrae (red asterisks in d). T2WI intermediate (e): enhancing extraosseous soft tissue mass (d and f) within the spinal canal extending into bilateral lateral recesses and neural foramina surrounding the bilateral traversing L1 and exiting T12 nerve roots (red arrows). T1WI, T1-weighted image; T2WI, T2-weighted image; STIR, short tau inversion recovery

Abnormal marrow signals were also appreciated in T12-L1 vertebral bodies, appearing hypointense signal on T1-weighted images (T1WI), isointense on T2-weighted images (T2WI), and hyperintense on short tau inversion recovery (STIR) images. Hyperintense signal was seen on T2WI in the intervertebral disc, protruding into the inferior endplate of the T12 vertebral body, suggesting discitis. Post-gadolinium T1WI sequences showed heterogeneous enhancement of T12 and L1 vertebrae with enhancing extra-osseous soft tissue mass within the epidural space of the spinal canal, extending into bilateral lateral recesses and neural foramina, surrounding the bilateral traversing L1 and exiting T12 nerve roots (Figure 2).

This intra-spinal epidural soft tissue had a mass effect on the conus medullaris. However, there was no intramedullary T2WI hyperintense signal to suggest myelopathy. No para-spinal fluid collection was seen. Cerebrospinal fluid (CSF) analysis was performed, which was initially inconclusive. CSF cultures for TB were also negative.

**Figure 3:**
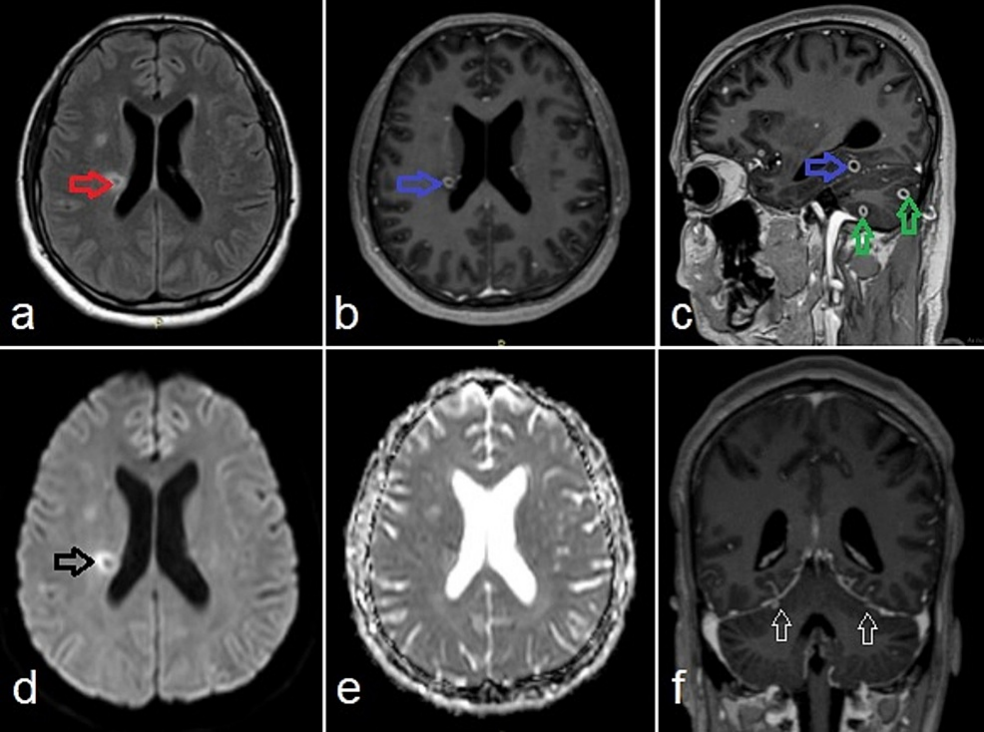
Brain MRI T1WI post-contrast axial (b) and sagittal (c) sections showing multiple ring-enhancing supra and infra-tentorial lesions (blue and green arrows, respectively) (b and c). Their wall appears hyperintense on FLAIR (red arrows in a). These show peripheral ADC (e) high signal (black arrows) with no central diffusion restriction (d) favoring tuberculomas. Furthermore, there is mild nodularity along the tentorium suggestive of meningeal involvement (white arrows in f). T1WI, T1-weighted image; FLAIR, fluid-attenuated inversion recovery; ADC, apparent diffusion coefficient

Subsequently, brain MRI with contrast was also performed that showed multiple intra-axial ring-enhancing supra and infra-tentorial lesions involving bilateral cerebral and cerebellar hemispheres and brainstem. These showed peripheral apparent diffusion coefficient (ADC) high signal with no central diffusion restriction, favoring tuberculomas. Furthermore, there was mild nodular leptomeningeal enhancement in the pre-pontine cistern, suggestive of meningeal involvement (Figure 3).

The CSF TB culture showed growth of Mycobacterium tuberculosis complex after six weeks, with sensitivity to streptomycin (1.00 µg/mL), isoniazid (0.10 µg/mL), rifampin (1.00 µg/mL), ethambutol (5.00 µg/mL), and pyrazinamide (100.0 µg/mL).

The patient was managed with tramadol and pregabalin for pain control and anti-tuberculous treatment (rifampicin, isoniazid, ethambutol, and pyrazinamide) with significant symptomatic improvement. He has recently completed one year of anti-tuberculous therapy and has been asymptomatic until the last follow-up.

## Discussion

CNS TB is a rare manifestation of extra-pulmonary TB. It occurs in only around 10% of patients with systemic TB. It commonly occurs in immunocompromised individuals such as those with HIV infection or on immunosuppressive therapy [3].

Meningitis is the most common form of CNS TB. It is mostly due to the hematogenous spread of bacteria; however, it can also occur due to the extension of subpial or subependymal focus (Rich focus) to intra- or extra-axial CSF spaces [2]. The most important MRI feature consists of diffuse leptomeningeal enhancement with distinctive predilection to involve the basal cisterns and extension along the inferomedial surfaces of the frontal lobes, the anteromedial surface of the temporal lobes, the floor of the third ventricle, the superior aspect of the tentorium cerebelli, and along the Sylvian fissures. In addition to this, altered CSF signal intensity is also appreciated [1,4]. This pattern of meningeal enhancement is also seen in infective meningitis, rheumatoid arthritis, sarcoidosis, and carcinomatous meningitis. Complications include progressive hydrocephalus, vasculitis, infarction, and cranial neuropathies [2].

Tuberculomas are the most common form of parenchymal lesions seen in CNS TB. Spinal involvement is rare, seen in 2% of all CNS TB cases and 0.2-5% of all CNS tuberculomas [5]. The ratio of intramedullary tuberculoma to intracerebral tuberculoma is approximately 1:42, and 72% of lesions are located in the thoracic cord [6]. The differential for ring-enhancing lesions includes neurocysticercosis, metastasis, CNS lymphoma (immunocompromised individuals), toxoplasmosis, tumors (astrocytoma, ependymoma, and hemangioblastoma), and pyogenic abscesses. Tuberculomas may resolve completely, but mostly they undergo calcification with the resolution of perilesional edema. They appear hypointense on T1WI and T2WI with no enhancement. Gradient recalled echo/susceptibility-weighted imaging (GRE/SWI) sequences show susceptibility artifact [1,6]. In the acute phase, symptoms of intramedullary spinal tuberculoma may predominate. MRI of the brain should be performed in all such cases so that prompt treatment can be initiated to prevent complications such as seizures and intracranial hypertension [7].

Other forms of parenchymal TB include cerebritis, cerebral abscess, miliary TB, or tuberculous encephalopathy. TB cerebritis or abscess may appear similar to that of pyogenic infection. Miliary form of disease is seen in severely immunocompromised patients with multiple tiny (2-3 mm) scattered lesions located at the corticomedullary junction. These may be invisible on non-contrast MRI or appear hypointense on T2WI and show ring enhancement. Tuberculous encephalopathy usually affects children who present with convulsions or coma. MRI shows cerebral edema and demyelination seen as hyperintensity on T2WI [2].

Tuberculous spondylitis, also known as “Pott’s Disease,” is the commonest form of extra-pulmonary TB. It commonly involves the thoracic spine followed by the lumbar spine. Typically, it involves multiple vertebral bodies sparing the intervertebral disc in the early stage. Para-spinal extension can occur with the formation of para-vertebral abscess [8]. The differential diagnosis of tuberculous spondylitis includes pyogenic and fungal infections, sarcoidosis, metastasis, and lymphoma. Although there are no pathognomonic features associated with Pott’s disease, long history, slow progression, and presence of large collections favor tuberculous spondylitis. Infectious spondylitis usually involves the intervertebral disc, while it is spared in case of TB [9]. Complications include subdural/epidural abscess that may result in spinal cord compression. MRI enables anatomic localization and extent, enables early detection, and thereby prevents severe spinal and neurological complications [8].

## Conclusions

CNS TB has a wide spectrum of patterns, including meningitis and parenchymal forms. Osseous involvement in the form of spondylodiscitis may also have serious neurological sequelae. Imaging along with clinical and pathological correlation enables accurate characterization of myriad forms of CNS TB in order to initiate prompt treatment and thereby prevent the morbidity and mortality of this disease.
